# Better Training for Matrons

**Published:** 1909-01-30

**Authors:** 


### Nursing Administration.

### BETTER TRAINING FOR MATRONS.

### IV.—DRUDGERY OR ADMINISTRATION.

There is much misconception in regard to the possibilities of becoming acquainted with the administrative duties of the matron. It is too often assumed that the mysteries of household management, of nursing organisation, and of the commissariat can only be revealed to the initiated, and the result of this is that the matron often has to find them out for herself, aided at best by the kindly advice of some experienced friend, when she is appointed to deal with them as head of a training school. Rightly considered, these matters are as much within the scope of training as the art of nursing, and no dislocation of system would be brought about were they taught as consistently as other subjects in hospitals of sufficient size to admit of each department of work being ordered on distinct lines. Just as the theoretical preparation of the matron for her duties can best be carried out in the training of junior probationers, supplemented by private study, fitting in with the work which has to be accomplished in the training school, so administrative instruction can best be carried out by making use of the regular hospital routine, and will be found sufficient if used in the right way to insure the efficiency of every student. It is not every probationer who welcomes the opportunity of gaining insight into hospital management. Indeed the matron in some hospitals finds her nurses most reluctant to leave the ward work to act as assistant in the linen room or kitchen. She is tempted to select for these subordinate posts probationers who are not conspicuously bright in their work, and whose services in the wards will not be greatly missed. In old days dull probationers were often kept for quite an undue proportion of their time "serving tables" as a happy expedient, since in counting out linen or making lists for the housekeeper their capacity for making blunders was no longer a matter of life and death. And these posts still retain a certain element of boredom. It is in such work, however, that the future matron will find her opportunity for learning her business. It needs but the turn of a hand to convert the subordinate administrative posts into a valuable and coveted part of the higher students' course. It is obvious that in training people to be matrons the hospital cannot be perpetually dislocating all its arrangements in order to give each candidate an opportunity for assuming the reins. Excellent as it may be for a woman who aspires to rule to try her hand at directing one department after another, no hospital could possibly stand the strain of having always a new student at the head of the housekeeping or the office, nor could any matron possibly find time to supervise a succession of these enthusiastic beginners. It is essential for the process of turning out well instructed students that they should work under the direction of capable heads of departments, getting a thorough ground knowledge of all the details and working through them in the same spirit of patient subordination which they bring to the study of nursing. If the head of each department knew how to teach, the time spent in what is now mere routine would be employed to lasting advantage. How much does the ordinary probationer who spends three months in the linen department know about the hospital linen? She masters the day's routine fairly quickly, and can cope with outgoings and incomings. She is familiar with lists and entries, and is a storehouse of information with regard to the contents of certain cupboards. But test her general grasp of the subject with a paper at the end of her term, and the limitations of her knowledge become painfully apparent. Has she formed any conception of the proper equipment as regards linen of medical and surgical wards respectively per bed? Could she make a complete list from memory of the articles required in the theatre? Has she studied the best materials to buy for the various purposes of the ward? Can she distinguish good sheeting from bad, and has she a knowledge of the contract prices and the difference which an extra penny a yard will make in the expenditure for the year and in the life of the sheet? All these things and many other branches of the subject ought to be brought under the notice of the student during her term of service in the linen department and the same holds good in all the work outside the ward which falls to her lot. She should be aided to envisage each department as a whole, and to study it until she has completely mastered not merely the intricacies of practice, but the principles of theory. Very little extra trouble would be caused by this intelligent use of opportunity. When contracts for linen, for instance, were to be made the student would be called in by the matron to study the materials, to state her reasons for choosing one rather than another, and to learn the reasons which guided the final choice. She would do her daily spell of routine work always with the consciousness of the importance of the whole. She would fill her notebook, under the sister's guidance, with valuable data for future reference, and she would have her faculties always sharpened by the remembrance that in her final examination half a dozen questions on the working of this particular department awaited her. The consequences of this method of work would be that on leaving the hospital the nurse would be fully prepared to organise the linen department wherever she might happen to be sent, and to deal with it successfully on the largest or smallest scale—in a sanatorium, a training school of major importance, or a base hospital of the Territorial Force. In the arrangement of such a practical curriculum as is here faintly indicated there is less show on paper than in a collegiate course, but every administrator will realise its superiority over any course which could be organised outside the institution. For by this means no break would occur in the probationer's course, and her training in other directions would suffer no interruption.

